# Validating a Scoring System for the Diagnosis of Smear-Negative Pulmonary Tuberculosis in HIV-Infected Adults

**DOI:** 10.1371/journal.pone.0095828

**Published:** 2014-04-22

**Authors:** Isabella Coimbra, Magda Maruza, Maria de Fátima Pessoa Militão Albuquerque, Joanna D’Arc Lyra Batista, Maria Cynthia Braga, Líbia Vilela Moura, Demócrito Barros Miranda-Filho, Ulisses Ramos Montarroyos, Heloísa Ramos Lacerda, Laura Cunha Rodrigues, Ricardo Arraes de Alencar Ximenes

**Affiliations:** 1 Post-Graduation Program in Tropical Medicine – Federal University of Pernambuco, Recife, Pernambuco, Brazil; 2 Aggeu Magalhães Research Center/Fiocruz, Recife, Pernambuco, Brazil; 3 Department of Clinical Medicine, University of Pernambuco, Recife, Pernambuco, Brazil; 4 London School of Hygiene and Tropical Medicine, London, United Kingdom; University of California, Davis, United States of America

## Abstract

**Background:**

The challenge of diagnosing smear-negative pulmonary TB (tuberculosis) in people living with HIV justifies the use of instruments other than the smear test for diagnosing the disease. Considering the clinical-radiological similarities of TB amongst HIV-infected adults and children, the proposal of this study was to assess the accuracy of a scoring system used to diagnose smear-negative pulmonary TB in children and adolescents, in HIV-infected adults suspected of having smear-negative pulmonary TB.

**Methods:**

A Phase III validation study aiming to assess the diagnostic accuracy of a scoring system for diagnosing smear-negative pulmonary TB in HIV-infected adults. The study assessed sensitivity, specificity, positive and negative likelihood ratios, and positive and negative predictive values of the scoring system. Three versions of the scoring system were tested.

**Results:**

From a cohort of 2,382 (HIV-infected adults), 1276 were investigated and 128 were diagnosed with pulmonary TB. Variables associated with the diagnosis of TB were: coughing, weight loss, fever, malnutrition, chest X-ray, and positive tuberculin test. The best diagnostic performance occurred with the scoring system with new scores, with sensitivity = 81.2% (95%-CI 74.5% –88%), specificity = 78% (75.6% –80.4%), PPV = 29.2% (24.5% –33.9%) and NPV = 97.4% (96.4% –98.4%), LR+ = 3.7 (3.4–4.0) and LR− = 0.24 (0.2–0.4).

**Conclusion:**

The proposed scoring system (with new scores) presented a good capacity for discriminating patients who did not have pulmonary TB, in the studied population. Further studies are necessary in order to validate it, thus permitting the assessment of its use in diagnosing smear-negative pulmonary TB in HIV-infected adults.

## Introduction

Tuberculosis (TB) is the most frequent cause of death in people living with HIV (PLHIV) [1], with high mortality in smear-negative cases [2]. Early diagnosis of smear-negative pulmonary TB in this group, if specific treatment is initiated quickly, has a favorable impact on mortality [3], [4]. However, this diagnosis remains a major challenge for public health [5].

Sputum smear microscopy (SSM) is the main method used for diagnosing pulmonary TB, especially in countries with limited financial resources [6], [7] although it fails to detect up to 50% of TB cases in PLHIV [5]. While this method achieves high specificity, sensitivity is not high, especially in the diagnosis of paucibacillary or extra-pulmonary forms of the disease [8]. These findings are frequent [9] and have been on the increase in PLHIV [10]. A negative smear may lead to either a late or even a lack of diagnosis [2], [3]. Furthermore, performing a sputum culture requires the necessary infrastructure, which is not always easily available in TB high-burden countries and, depending on the method used, results may take up to weeks to become available [8].

Pulmonary TB in HIV-infected adults may appears with a post-primary or typical radiological pattern in the early stages of infection or with an atypical or primary radiological pattern in more advanced stages of HIV infection [11], [12]. Immunocompromised adults present a TB profile similar to that encountered in children [13]. With children, sensitivity to sputum culture or gastric lavage is low, 30–40%, due to both technical difficulties and the pathogenesis of the disease in this group. Paucibacillary tuberculosis is more common in this age group [14], [15].

Scoring systems constitute an alternative tool for the diagnosis of pulmonary tuberculosis in situations where there is a negative sputum smear test [16]. The purpose of such systems is to quantify the probability of a particular event based on previously identified criteria [17]. The Brazilian Ministry of Health has adopted a scoring system for the diagnosis of childhood/adolescent, smear-negative TB [18]. It uses clinical and radiological features and tuberculin skin test reactivity, to which scores are assigned arbitrarily. This scoring system was validated for children aged under 15 years, undergoing either outpatient [14], [19], [20] or inpatient investigation [14], [21]. Because of the similarities in the clinical and radiological presentations of TB in children and many HIV-infected adults with TB, the purpose of the present study was to assess, among TB suspects, the accuracy of this scoring system for the diagnosis of smear-negative pulmonary TB in HIV-infected adults, and also to assess the association between clinical, radiological and immunological factors and the diagnosis of tuberculosis within this group.

## Methods

### Ethics Statement

This study is part of the CSV Project 182/06 - Project for clinical and epidemiological study of TB/HIV co-infection in Recife, approved by the Ethics Committee of the Universidade Federal de Pernambuco (registration SISNEP FR-067 159/CAAE 0004.1.172.106-05/register CEP/CCS/UFPE 254/05).

### Study Design

The present study was composed of four stages. The first stage corresponded to a Phase III validation study [22] of a scoring system adapted for diagnosing pulmonary TB in HIV-infected adults, with negative or unperformed smear tests [Adapted Scoring System(ASS)]. All enrolled individuals were suspected of having tuberculosis and the objective of the study was to evaluate if, among suspects, the scoring system would distinguish those with and without the target disorder (tuberculosis). Phase III studies differ from Phase I and II studies because, in the latter, the comparison is made between a group of patients who already have a established diagnosis and normal individuals [22].

In the second stage of the study, a case-control analysis was undertaken, in which cases were considered as those individuals with pulmonary TB, and non-cases (controls) were pulmonary TB suspects, but undiagnosed with TB. The association between pulmonary TB and different groups of variables potentially associated with the disease was assessed. This step aimed to identify variables, which could be added to the scoring system. In the third stage, the accuracy parameters for the model identified in the previous stage [Modified Scoring System(MSS)] were calculated. The fourth stage proposed another scoring system [Scoring System with New Scores(SSNS)]. To propose a scoring system with new scores we used the values of the coefficients β estimated in the final multivariate logistic model (following the example of other authors [17], [23], [24] from the second stage. In the logistic model these coefficients are obtained using the maximum likelihood method and they represent the change in the log odds that would result from a one unit change in one variable when all others are fixed. The coefficient β of each variable was multiplied by 10, in order to optimize the rounding off. Subsequently, accuracy parameters were calculated. The analysis was repeated limiting the total score to 15 to evaluate if there would be a if loss of accuracy as such a simplified version, that could be easily used without a calculator, could potentially increase the user-friendliness and utilization/uptake of the scoring system in practice.

### Study Population

Individuals aged over 18 years enrolled on a cohort of HIV-infected adults from July 2007 to December 2010 and who were pulmonary TB suspects. These individuals attended two referral centers for HIV treatment in the state of Pernambuco, in the Northeastern of Brazil. Pernambuco is the Brazilian state with the second highest TB mortality rate (4.0 deaths per 100,000 inhabitants per year), almost double as compared to the national rate [25]. The two referral centers serve approximately 70% of all HIV cases in the state. Patients were monitored for a minimum period of six months.

### Definition of Terms and Study Variables

Individuals were considered pulmonary TB suspects if they complained of cough (with or without sputum production) for any length of time, and/or weight loss with a negative or unperformed smear test. The choice of criteria was based on Cain *et al.* [26] and Koole *et al.* [10] and on the WHO publication [8], which suggests that cough alone for two weeks or more is an insensitive criterion for the diagnosis of tuberculosis in PLHIV. An association of symptoms is often necessary for such a diagnosis in this group but the most appropriate combination remains unknown [8].

**Figure 1:**
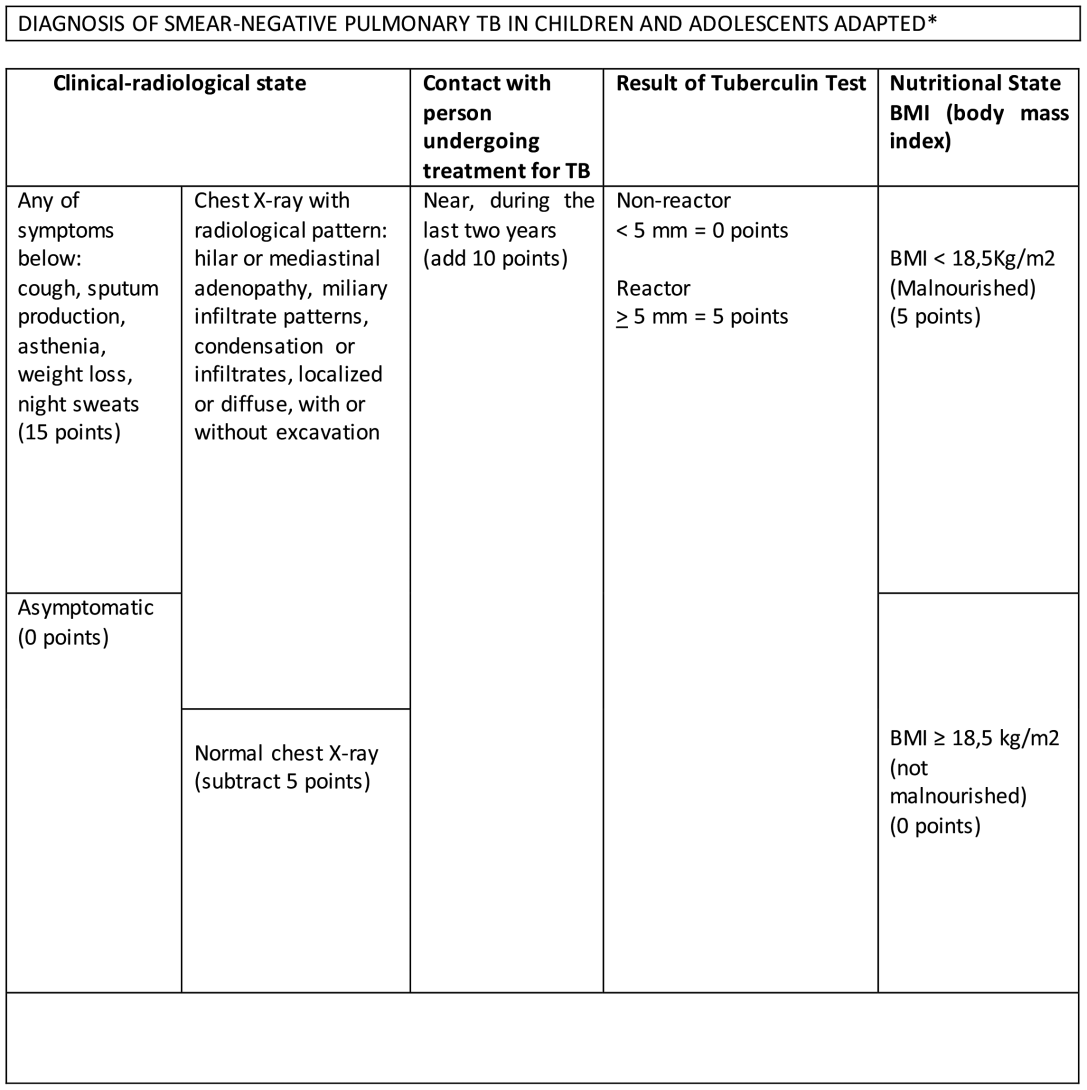
Diagnosis of smear-negative pulmonary TB in children and adolescents adapted.* *scoring system for diagnosing smear-negative pulmonary TB HIV-infected adults, adapted from the scoring system to diagnose smear-negative pulmonary TB in children and adolescent. Source: *Ministério da Saúde. Tuberculose. Guia de Vigilância Epidemiológica, Brasil, 2002*.

The main problem to evaluate a scoring system for diagnosing tuberculosis in PLHIV is the choice of gold standard [1]. Although a perfect gold standard is seldom available in diagnostic research, this problem is particularly challenging when diagnosing tuberculosis in this group. In the absence of a perfect gold standard, individuals in the present study were considered pulmonary TB cases if (1) they presented a positive sputum culture for *M. tuberculosis* or (2) had been notified as having pulmonary TB to the Brazilian National System for Surveillance and Control of Diseases (SINAN) or to the Mortality Information System (SIM) during the 6-month period after the interview or even, (3) those individuals without bacteriological confirmation, with clinical-radiological improvement after treatment for TB was initiated by the attending physician, in a follow-up period of no less than 6 months. All patients were followed-up for at least six months using the information from medical records, SINAN and SIM. The follow-up procedure was aimed at retrospectively assessing the health status at time zero, instead of ascertaining the reference standard of diagnosis for tuberculosis at time zero itself. The scoring system tested was that recommended by the Brazilian Ministry of Health for diagnosing childhood/adolescent TB [18], adapted for HIV-infected adults with suspected smear-negative pulmonary TB (ASS). The adapted criteria are presented in Figure 1.

Factors potentially associated with pulmonary TB and analyzed in the second, third and fourth stages will only be commented upon should they call for any further clarification. The radiological patterns were categorized as typical (cavitations or infiltrates in the upper lobes of the lungs), atypical (pleural effusion, hilar or mediastinal lymph nodes, diffuse or restricted infiltrates in the lower lobes) and scars (fibrosis in the summits and upper lobes of the lungs and calcified nodules). Alcohol consumption was categorized as: abstainer/light drinker (has never drunk or drinks two days per week, but not exceeding 10 drinks per month), moderate drinker/heavy (drinks at least 3–4 days per week, exceeding 5 drinks per day or undergoing treatment for alcoholism). Smokers were categorized as: nonsmokers (those who have never smoked during their lifetime), former smokers (those who stopped smoking at least six months prior to study entry) and smokers (those who were smokers at the time of enrollment or had stopped smoking during the six months prior to study entry). The criteria used to define AIDS were those adopted by the Brazilian Ministry of Health [27]. The drug use variable was analyzed assessing each of the drugs independently (marijuana, cocaine, crack and glue). Antiretroviral therapy (ART) was defined as a combination of three different antiretroviral drugs, regardless of the number of drug classes used. The tuberculin test (TT) was considered positive or reactive with a measurement of induration ≥5 mm, according to the parameters defined for PLHIV [18]. The CD4 variable was categorized as ≥200 cells/mm3 and <200 cells/mm3, as the latter reflects a greater immunological impairment and is associated with increased mortality in PLHIV [6], [28].

### Data Collection

Patients attending the two referral services were invited to participate in the cohort. They were interviewed by previously-trained health professionals, using standardized questionnaires, after signing the informed consent. Additional information was collected from medical records. Each patient was required to undertake a chest X-ray, a blood count, a CD4 count and viral load determination, TT, sputum microscopy and culture for mycobacteria (for those who presented with sputum production) in accordance with Brazilian Ministry of Health norms [18]. Brazilian Ministry of Health administration and reading techniques were adopted for TT [18]. Radiologists from the above-mentioned hospitals produced the radiology reports, according to previously defined standards, with masking in relation to the clinical suspicion of pulmonary tuberculosis. Individuals with suspected pulmonary TB were included in the study.

### Statistical Analysis

To analyze the accuracy of the ASS for the diagnosis of tuberculosis in HIV-infected adults, a receiver operating characteristic (ROC) curve was constructed and sensitivity (Se), specificity (Sp), positive (PPV) and negative (NPV) predictive values, positive (LR+) and negative (LR−) likelihood ratios, with their respective confidence intervals, were calculated.

For the second stage of the research, independent variables were grouped into blocks: biological variables (gender, age); clinical variables (fever, weight loss, coughing, sweating, asthenia, sputum production, hemoptysis, chest pain, peripheral lymphadenopathy, malnutrition (body mass index (BMI) <18 kg/m^2^); variables related to habits and lifestyle (smoking, drinking and illicit drugs use); variables related to HIV (AIDS case, CD4 count, viral load, ART); epidemiological variables (contact with TB, past TB); variables related to diagnostic methods for TB (radiologic pattern, which was defined as normal, typical or atypical and scar; TT presented as reactive or non-reactive); history of cancer and use of systemic corticosteroids.

The magnitude of association with the diagnosis of TB was measured by odds ratio (OR) and statistical significance was tested by the confidence interval (95%-CI) and *p* value (chi-square test or maximum likelihood ratio). Significance level was set at *p*<0.05. The multivariate logistic regression model was conducted in two steps. In the first, all variables that comprised the ASS were introduced into the model, and those with a *p*-value <0.05 in association with the outcome remained in the model. In the second step, HIV-related variables that showed a *p*<0.25 in the univariate analysis were introduced into the model, and those with *p*<0.05 remained in the model.

In the third stage a score was given to the HIV-related variables that remained in the multivariate model, and the accuracy parameters of the MSS were calculated. In the fourth stage, so as to create a scoring system with a new set of scores, the variables that remained in the final multivariate regression model were employed. By using the coefficient β derived from each independent variable of the model, a score was attributed to each variable, which was the coefficient β of each variable multiplied by 10, in order to optimize rounding off. The accuracy parameters of the SSNS were calculated.

The population diagnosis attributed percent (PDA%) –the fraction of diagnosis in the total study population attributable to a factor – was estimated for multiple factors according to Bruzzi *et al*. [29] for population attributable risks. Estimates were based on the adjusted odds ratio and on the proportion of diseased (tuberculosis) individuals presenting the factor under consideration. Evaluating the relevance of the factor to the diagnosis is of considerable assistance. It should be noted that when calculating the ensemble of variables, the estimatation is not equivalent to adding the PDA% to individual factors, because the approach controls for confounding. [29].

## Results

**Figure 2:**
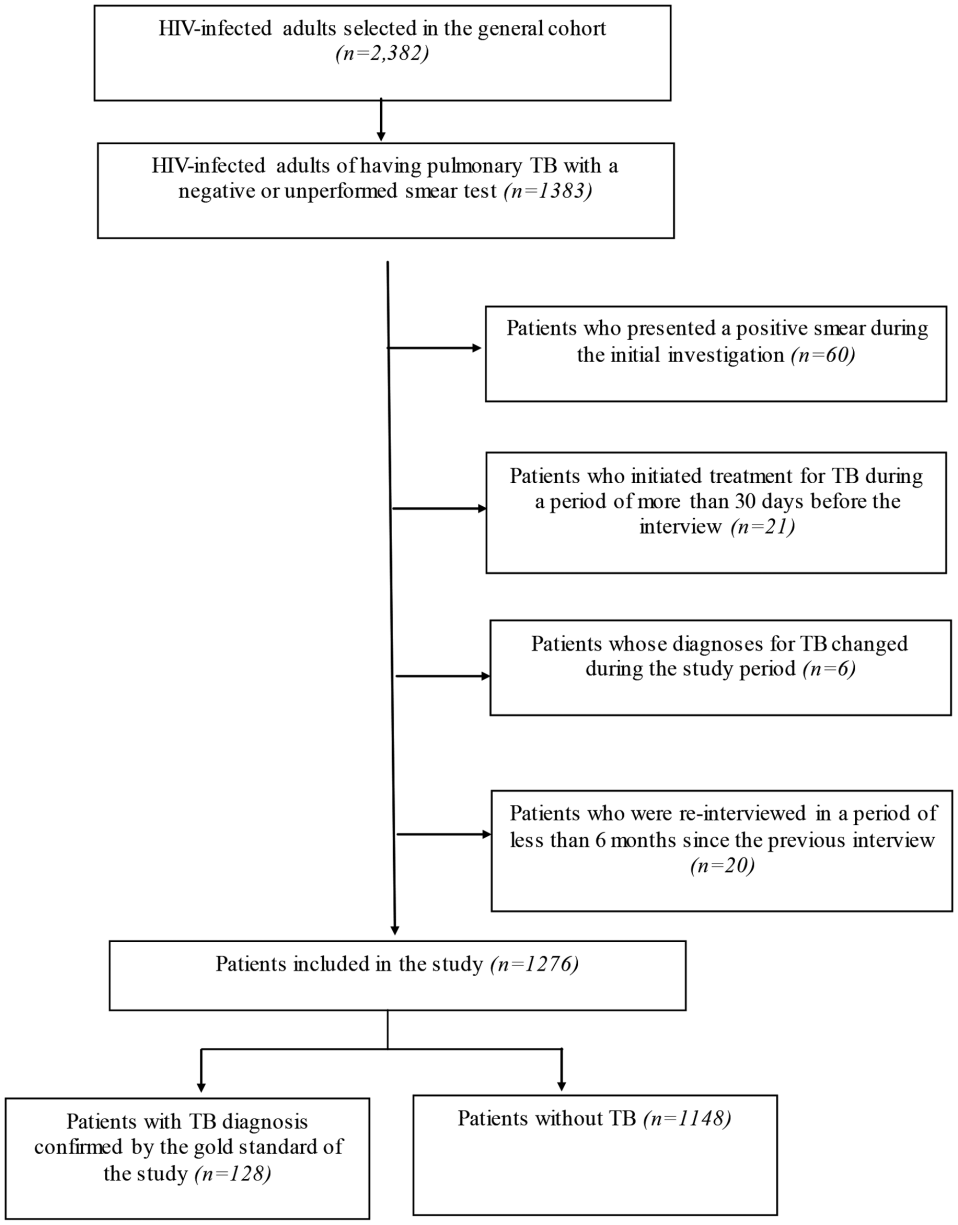
Algorithm of the selection of the study population to assess the accuracy of the scoring system in diagnosing smear-negative pulmonary TB in HIV-infected adults in Pernambuco, Brazil, 2007–2010.

From a cohort of 2,382 HIV-infected adults, 1383 were pulmonary TB suspects in accordance with the criteria defined in the present study. Of these, 107 met the exclusion criteria (Figure 2). Of the remaining 1276 individuals, 128 (10%) were diagnosed with the pulmonary form of tuberculosis (5 presented an association with the extra-pulmonary form, and 3 with the disseminated form of the disease), during the period of the study.

Of the total number of studied individuals, 747 (58.6%) were male and 201 (15.8%) were aged 30 years or less, 1039 (81.5%) were aged between 30 and 59 years and 35 (2.7%) were 60 years or over. Coughing was reported by 975 (76.5%) and weight loss by 664 (53.1%). Of those who had undergone a chest X-ray (n = 1043, 82.6%), 186 presented a typical/atypical radiological pattern and 129 a scar pattern, and of those who underwent the TT (n = 548, 43%), 464 were non-reactive and 84 reactive. The majority of patients were taking ART (n = 951, 74.8%) and 282 (26.6%) presented with CD4 counts <200 cells/mm^3^.

**Figure 3:**
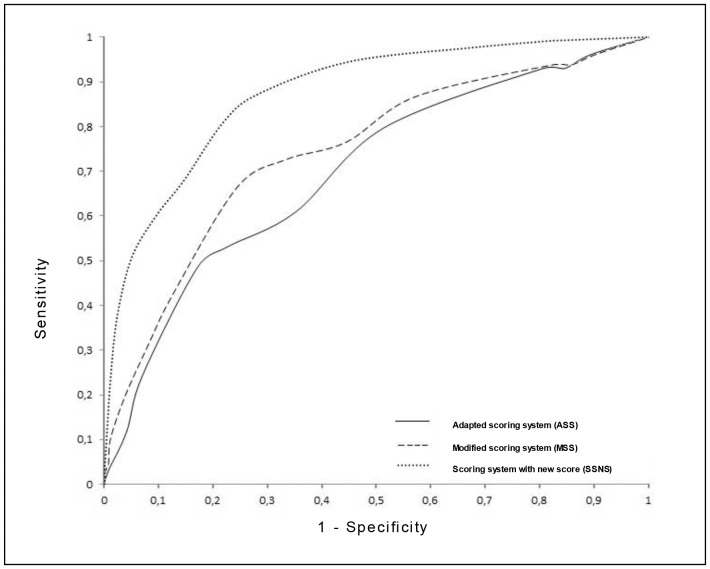
Comparison of ROC curves for the Adapted Scoring System (ASS), Modified Scoring System (MSS) and Scoring System with New Scores (SSNS) for the diagnosis of smear-negative pulmonary TB in HIV-infected adults.

Assessment of the ASS for HIV-infected adults through the ROC curve indicated an area under the curve of 0.6960 (95%-CI 0.670–0.721) (Figure 3). An optimized diagnosis cut-off value ≥20 points was obtained (Table 1), with the following results: Se = 60.9% (52.5%–69.4%), Sp = 64.7% (61.9%–67.5%), PPV = 16.1% (12.9% –19.4%) and NPV = 93.7% (92% –95.4%), LR+ = 1.73 (1.5–2.0) and LR− = 0.60 (0.5–0.8). Variables that presented a significant association with the diagnosis of pulmonary TB in the univariate analysis were: - biological: male, - habits and lifestyle: abstainer, - clinical: coughing, weight loss, presence of sputum with or without blood, weight loss, fever, sweating, asthenia, BMI <18.5 kg/m2, lymphadenopathy; - related to diagnostic methods for TB: typical, atypical or scar pattern chest X-ray, reactive TT; - HIV-related: the absence of ART, CD4 count <200 cells/mm^3^. No association was obtained between cancer and use of systemic corticosteroids and the condition of the TB cases. Variables that remained in the final multivariate model are shown in Table 2.

**Table 1:** Values of sensitivity, specificity, positive and negative predictive values, positive and negative likelihood ratios for Adapted Scoring System (ASS), Modified Scoring System (MSS) and Scoring System with New Score (SSNS) according to the cut-off values (with the optimized cutoff value marked in “bold-face”), for HIV-infected adults and suspected smear-negative pulmonary TB, in Pernambuco, Brazil, 2007–2010.

Number of points	Sensitivity	Specificity	Predictive value			Likelihood Ratio	
			Positive	Negative		Positive	Negative
**Adapted Scoring System (ASS)**							
≥ −5	100%	0%	10.04%	–		1.000	–
≥0	96.09%	10.5%	10.70%	96.01%		1.074	0.370
≥5	92.97%	15.34%	10.91%	95.13%		1.098	0.458
≥10	92.97%	19.35%	11.40%	96.10%		1.153	0.363
≥15	79.69%	48.56%	14.74%	95.54%		1.549	0.418
** ≥20**	**60.94%**	**64.69%**	**16.15**%	**93.68**%		**1.726**	**0.604**
≥25	53.13%	77.24%	20.66%	93.65%		2.335	0.607
≥30	48.44%	82.82%	23.93%	93.50%		2.820	0.622
≥35	24.22%	92.94%	27.68%	91.65%		3.429	0.815
≥40	11.72%	95.82%	23.83%	90.67%		2.800	0.921
≥45	3.13%	99.13%	28.64%	90.17%		3.584	0.977
≥50	0%	100%	–	89.96%		–	1.000
**Modified Scoring System (MSS)**							
≥ −5	100%	0%	10.04%	–		1.000	–
≥0	96.09%	9.85%	10.63%	95.76%		1.066	0.396
≥5	93.75%	14.30%	10.88%	95.34%		1.094	0.437
≥10	93.75%	17.96%	11.31%	96.26%		1.143	0.348
≥15	86.72%	42.81%	14.47%	96.65%		1.516	0.310
≥20	76.56%	55.54%	16.12%	95.50%		1.722	0.422
≥25	72.66%	66.61%	19.54%	95.62%		2.176	0.410
≥**30**	**66.41%**	**75.68%**	**23.35**%	**95.28**%		**2.730**	**0.444**
≥35	42.19%	87.62%	27.55%	93.14%		3.408	0.660
≥40	31.25%	91.98%	30.30%	92.30%		3.896	0.747
≥45	19.53%	96.34%	37.32%	91.47%		5.334	0.835
≥50	10.16%	98.95%	51.92%	90.80%		9.708	0.908
≥55	4.69%	99.30%	42.78%	90.32%		6.721	0.960
≥60	3.13%	99.83%	67.26%	90.22%		17.922	0.970
>60	0%	100%	–	89.96%		–	1.000
**Scoring System with New Scores (SSNS)**							
≥0	100%	0%	10.04%	–		1.000	–
≥5	100%	0%	10.04%	–		1.000	0.000
≥10	100%	0.09%	10.05%	100%		1.001	0.000
≥15	99.22%	17.35%	11.81%	99.50%		1.200	0.045
≥20	99.22%	17.70%	11.86%	99.51%		1.205	0.044
≥25	97.66%	32.35%	13.87%	99.20%		1.443	0.072
≥30	94.53%	55.01%	19.00%	98.90%		2.101	0.099
≥35	87.50%	71.40%	25.45%	98.08%		3.060	0.175
** ≥40**	**81.25%**	**78.03%**	**29.21**%	**97.39**%		**3.698**	**0.240**
≥45	67.97%	85.44%	34.25%	95.98%		4.668	0.375
≥50	60.16%	90.50%	41.41%	95.32%		6.330	0.440
≥55	50.78%	95.03%	53.27%	94.54%		10.219	0.518
≥60	37.50%	97.73%	64.83%	93.34%		16.543	0.639
≥65	23.44%	98.95%	71.36%	92.05%		22.402	0.774
≥70	14.06%	99.48%	75.11%	91.21%		26.883	0.864
≥75	5.47%	99.91%	87.15%	90.45%		62.729	0.946
≥80	3.91%	100%	100%	90.31%		44.806	0.962
>80	0%	100%	–	89.96%		–	1.000

Sample size = 1276 individuals.

**Table 2:** Multivariate analysis of the factors associated to the diagnosis of pulmonary TB, and the scoring for each variable, in HIV-infected adults and suspected smear-negative pulmonary TB, in Pernambuco, Brazil, 2007–2010.

Variables	OR (95%-CI)	*p*-value	β	New score*	% PDA%***
**Model**					**68.2**
** Clinical Symptoms**					**88.4**
Cough**					
No	1.0	–		0 points	
Yes	3.96 (1.95–8.05)	0.000	1.38	14 points	66.0
Weight loss					
No	1.0	–		0 points	
Yes	2.44 (1.43–4.15)	0.001	0.89	9 points	43.9
Fever					
No	1.0	–		0 points	
Yes	1.99 (1.22–3.23)	0.005	0.69	7 points	25.3
Malnutrition					
No	1.0	–		0 points	
Yes	2.62 (1.51–4.54)	0.001	0.96	10 points	18.6
**Related to diagnostic methods for TB**					**62.0**
Radiological pattern					
Normal	1.0	–	–	0 points	
Typical/Atypical	9.14 (5.33–15.7)	0.000	2.21	22 points	
Scar	2.81 (1.32–5.96)	0.007	1.03	10 points	59.4
No X-ray	0.93 (0.43–2.01)	0.851	–		
Tuberculin test					
Non-reactor	1.0	–	–	0 points	
Reactor	4.03 (1.63–9.98)	0.003	1.39	14 points	6.5
Not undertaken	2.64 (1.49–4.70)	0.001	0.97	10 points	
**Related to HIV**					**42.1**
CD4 T cell count					
≥200	1.0	–	–		
<200	3.19 (1.82–5.57)	0.000^†^	1.16	12 points	42.1

*To optimize the rounding off of the score, the regression scores were multiplied by 10.

**For people living with HIV and suspected smear-negative pulmonary TB, whose criteria on entering the study was weight loss.

*** Population diagnosis attributed percent - percent of diagnosis attributed to a factor or group of factors.

Sample size 1276 individuals.

For the MSS, which included CD4 count, the area under the curve was 0.743 (95%CI-0.781–0.767) (Figure 3). The best cut-off value was ≥30 points (Table 1), with the following parameters: Se = 66.4% (57.5% –74.5%), Sp = 75.7% (73.1% –78.1%), PPV = 23.4% (9.1% –28.0%) and NPV = 95.3% (93.7% –96.6%), LR+ = 2.73 (2.4–3.1) and LR− = 0.44 (0.3–0.6).

The assigned values in points, for each variable of the proposed scoring system, together with the respective coefficients β are detailed in Table 2. The area under the curve was for the SSNS and was 0.8751 (95%-CI 0.8438–0.9063) (Figure 3). A better diagnostic performance was registered for a cut-off value of 40 (Table 1), with Se = 81.2% (74.5% –88%), Sp = 78% (75.6% –80.4%), PPV = 29.2% (24.5% –33.9%) and NPV = 97.4% (96.4% –98.4%), LR+ = 3.7 (3.4–4.0) and LR− = 0.24 (0.2–0.4).

The PDA% estimated for each variable in the final multivariate model ranged from 6.5% to 66.0%, and the PDA% for a combination of all factors was 68.2%.

The results of the simplified version, limiting the total score to 15 are presented in Tables 1 and 2. Table 1 shows the multivariate analysis of the factors associated with the diagnosis of pulmonary TB and the score assigned to each factor in the simplified version. Table 2 compares the accuracy parameters of the SSNS according to the cut-off values (with the optimized cut-off value marked in “bold-face”), with those of the simplified version. A loss of discriminatory power was observed.

## Discussion

The scoring system of the Brazilian Ministry of Health for the diagnosis of pulmonary TB in children and adolescents was adapted by the authors of the present study for HIV-infected adults (ASS), presenting with suspected smear-negative pulmonary TB, and when tested, presented with a cut-off value of 20 points, sensitivity of 60.9%, specificity of 64.7%, and LR+ = 1.7 and LR− = 0.6. The performance of the ASS in the study population fell below that of the Brazilian Ministry of Health scoring system with the population for which it was originally purposed. Studies that used this score system for diagnosing TB in children and adolescents reported sensitivity of 89% to 92% and specificity of 70% to 86% with 30 points [19], [14], in agreement with the results initially described when assessing the accuracy of the test [30]. In the univariate analysis, all the variables that make part of the ASS presented a statistically significant association with the diagnosis of TB. However, contact with TB did not remain in the final multivariate model. This is probably due to the presence of other variables that were associated with contact with TB, but that have a closer association with the diagnosis.

In the present study there was an association between the positive result of TT and the diagnosis of TB, although an association was also found between the unperformed TT and the diagnosis of TB. We have no explanation for this fact. It could be that the clinical features of these patients are more suggestive of TB or a more severe form of the disease, and the attending physician initiated treatment earlier. Although recommended by the Ministry of Health, the TT is not routinely used in these circumstances.

The variables that remained in the final model had already been considered in the Ministry scoring system except for the CD4 count <200 cells/mm^3^. The CD4 count was proposed because it is an important immunosuppression marker in HIV-infected patients [31] and the choice of the cut-off value (<200) was due to its association with more severe immune impairment [6]. Increased accuracy was observed when the CD4 count was included in the system (MSS), and an additional increase with the new proposed score (SSNS). Even with this increase, the probability of the disease in a positive individual was still low, around 29%. There was a high probability of an absence of disease (97.4%) in an individual with a negative result.

Other studies have also used a scoring system as a diagnostic tool for pulmonary TB in smear-negative TB suspects [7], [16], [23], [24], [32]. Of these, several were retrospective (7,23,32), thus constituting a limitation of these studies. Unlike the present study, no other study assessed a population of exclusively HIV-infected individuals. The measurements of accuracy varied considerably amongst the studies: sensitivity 70% to 94%, specificity 42% to 94%, PPV 43% to 73%, NPV 93% to 98.7% in localities with a different prevalence of TB. The LR was calculated and its usefulness discussed in a limited number of studies, with reports of 3.85 for LR+ and 0.36 for LR− in one study [24] and of 7.1 for LR+ and 0.2 for LR− in another [23]. However in the second study, the difference in the frequency of tuberculin testing between cases and controls, a variable strongly associated to diagnosing TB, may have caused selection bias and therefore, interfered with the encountered accuracy.

The accuracy of the proposed SSNS is similar to those observed in other studies, which used the same measurements, with the exception of Kanaya *et al* [23]. The population diagnosis attributed percent indicates that the studied factors explain approximately 68.2% of all diagnoses. The higher figure obtained for clinical symptoms highlights the importance of this subgroup of variables compared to those related to diagnostic methods for TB and CD4 counts.

The literature describes other types of instruments for diagnosing smear-negative TB in HIV-infected patients. The proposed diagnostic algorithm, updated by WHO [33], was recently assessed by several studies with different measures of accuracy: Koole *et al* [10](Se = 58.8%, Sp = 79.4%, PPV = 22.2% and NPV = 95.1%), Wilson *et al* [34] (Se = 80%, Sp = 44%, PPV = 34%, NPV = 86%, LR+ = 1.43 and LR− = 0.46), and Walley *et al* [2] (Se = 96%, Sp = 98%, PPV = 93% and NPV = 99%). This latter study with improved accuracy, notes however that only 13% of the studied population completed all the elements of the algorithm, which is one significant limitation. In other studies, algorithms or a combination of clinical features, supplemented with chest X-rays and a CD4 counts were proposed [5], [26], [35]. The results of which varied from no association with the diagnosis of TB [35] to a greater variation in the measurements of sensitivity (64.5%–93%) specificity (35%–64%), and PPV (9.9%–24%), but not in the NPV (95%–97%) [5], [32]. Only one study included and discussed the LR calculations, with an interval of 1.44–1.76 for LR+ and of 0.18–0.27 for LR− [26]. Getahun *et al* [1], in a meta-analysis investigating TB in PLHIV, with no discrimination of the smear, proposed an investigation system with the presence of any 1 of 4 specific clinical criteria for investigating TB with Se = 78.9%, Sp = 49.6%, NPV = 95.3% for the combination. With the exception of Walley *et al* [2], the above-cited studies presented a diagnostic accuracy similar to that observed in the present study, in relation to the presented measurements of accuracy. The abovementioned studies indicate a great variation in accuracy measurements indicating that the diagnosis of smear-negative pulmonary TB in PLHIV remains an enormous diagnostic challenge, especially when diagnostic resources are limited.

The present study presents a number of limitations. One of them is how to define a case of TB, without bacteriological confirmation in most cases, using clinical and radiological improvement criteria after initiating treatment for TB. In the absence of a perfect gold standard the present study used a reference standard that would approximate the gold standard [36]. Furthermore, this criterion was also used in other studies that applied the scoring system [19], [20] as well as in a study with another scoring system [32]. The difficulty of obtaining material for bacteriological confirmation, delayed culture results and the impossibility of adopting more expensive, complex diagnostic methods, justify the use the therapeutic response as criteria for defining TB. The range of the scoring system proposed makes it more difficult to be used without a calculator. However the analysis of the simplified version, limiting the total score to 15, showed a loss of discriminatory power. One further limitation is related to the fact that the results encountered maybe be only valid for the population studied. As the discriminatory power of the point scoring system may vary across settings, due to differences in the frequency of co-infection and due to the variability of signs and symptoms among patients, it would be advisable to replicate this Phase III study, applying the SSNS to HIV-infected adults in different settings with different epidemiological scenarios.

Nevertheless, this study has some methodological strengths. This was a prospective study, thus minimizing information bias. Participants were both outpatients and inpatients, therefore avoiding the selection of individuals with a given potential of severity. All patients were followed up for a minimum period of six months in order to reduce the misclassification of cases and non-cases of TB. All subjects were HIV-positive, a fact not observed in other studies involving scoring systems.

## Conclusion

The accuracy of the Brazilian Ministry of Health scoring system when used for diagnosing smear-negative TB in HIV-infected adults limits its use in clinical practice. In the studied population, the new scoring system presented a similar accuracy to other instruments tested, and a good capacity for distinguishing patients without pulmonary TB. Further studies should apply this new scoring system in order to validate it, thus assessing its use in diagnosing pulmonary smear-negative TB in HIV-infected adults.
